# Serum glucose–potassium ratio predicts inhospital mortality in patients admitted to coronary care unit

**DOI:** 10.1590/1806-9282.20240508

**Published:** 2024-10-07

**Authors:** Fulya Avcı Demir, İbrahim Ersoy, Ahmet Şeyda Yılmaz, Gökay Taylan, Emin Erdem Kaya, Ertan Aydın, Muammer Karakayalı, Muhammed Mürsel Öğütveren, Aybike Taşdelen Acar, Şıho Hidayet

**Affiliations:** 1 Istinye University, Department of Cardiology – İstanbul, Turkey.; 2 Medical Park Hospital, Department of Cardiology – Antalya, Turkey.; 3 Kepez State Hospital, Department of Cardiology – Antalya, Turkey.; 4 Recep Tayyip Erdogan University, Faculty of Medicine, Department of Cardiology – Rize, Turkey.; 5 Trakya University, Department of Cardiology – Tekirdağ, Turkey.; 6 Ersin Arslan Training and Research Hospital, Department of Cardiology – Gaziantep, Turkey.; 7 Giresun University, Department of Cardiology – Giresun, Turkey.; 8 Kafkas University, Training and Research Hospital, Department of Cardiology – Kars, Turkey.; 9 Istanbul Cardiology Institute, Department of Cardiology – İstanbul, Turkey.; 10 Inönü University, Faculty of Medicine, Department of Cardiology – Malatya, Turkey.

**Keywords:** Coronary care unit, Inhospital mortality

## Abstract

**OBJECTIVE:**

The aim of our study was to determine the role of serum glucose–potassium ratio in predicting inhospital mortality in coronary care unit patients.

**METHODS:**

This study used data from the MORtality in CORonary Care Units in Turkey study, a national, observational, multicenter study that included all patients admitted to coronary care units between September 1, 2022, and September 30, 2022. Statistical analyses assessed the independent predictors of mortality. Two models were created. Model 1 included age, history of heart failure, chronic kidney disease, hypertension, diabetes mellitus, and coronary artery disease. Model 2 included glucose–potassium ratio in addition to these variables. Multivariate regression and receiver operating characteristic analysis were performed to compare Model 1 and Model 2 to identify if the glucose–potassium ratio is an independent predictor of inhospital mortality.

**RESULTS:**

In a study of 3,157 patients, the mortality rate was 4.3% (n=137). Age (p=0.002), female gender (p=0.004), mean blood pressure (p<0.001), serum creatinine (p<0.001), C-reactive protein (p=0.002), white blood cell (p=0.002), and glucose–potassium ratio (p<0.001) were identified as independent predictors of mortality through multivariate regression analysis. The receiver operating characteristic analysis indicated that Model 2 had a statistically higher area under the curve than Model 1 (area under the curve 0.842 vs area under the curve 0.835; p<0.001). A statistically significant correlation was found between the inhospital mortality and glucose–potassium ratio (OR 1.015, 95%CI 1.006–1.024, p<0.001).

**CONCLUSION:**

Our study showed that the glucose–potassium ratio may be a significant predictor of inhospital mortality in coronary care unit patients.

## INTRODUCTION

Cardiovascular diseases are a leading cause of mortality and morbidity worldwide. The demand for intensive cardiac care is increasing^1^. However, there is limited information available on mortality and morbidity rates, as well as predictors of patients in coronary care units (CCUs). Early prognosis prediction is crucial in the follow-up of CCU patients hospitalized with high-risk diagnoses, such as acute coronary syndrome (ACS), fatal arrhythmias, and cardiogenic shock. Identifying important determinants of mortality can improve management strategies and outcomes in the CCU^2^. However, a comprehensive analysis of factors influencing the survival of CCU patients is necessary. Recent research has focused on prognostic biomarkers, such as inflammatory biomarkers, immune parameters, and metabolic/homeostatic indices.

An acute stressful event activates the hypothalamus-pituitary-adrenal system, leading to the release of stress hormones. This neuroendocrine response also activates the sympathetic system, which results in a significant increase in serum glucose levels and a significant decrease in serum potassium levels through catecholamines^3,4^. These metabolic changes stimulate the release of proinflammatory and procoagulant factors. This subsequently causes an inflammatory response and oxidative stress^5^.

The glucose–potassium ratio (GPR) is a simple, cost-effective analysis that has been evaluated in various patient groups, including those with traumatic brain injury, subarachnoid hemorrhage, delayed neuropsychiatric syndrome, and ischemic stroke accompanied by systemic stress. It has been found to be significantly associated with morbidity and mortality in all of these groups^6–10^.

In this study, we aimed to investigate the effect of GPR levels on mortality in CCU patients in Turkey using data from the MORtality in CORonary Care Units in Turkey (MORCOR-TURK) Study.

## METHODS

### Study design

The MORCOR-TURK Trial, a multicenter, prospective, cross-sectional, non-interventional registry study, was registered on clinicaltrials.gov with the number NCT05296694.

The study includes all consecutive patients admitted to the CCU in 50 cardiology centers from 7 geographical regions for a period of 1 month. The predictors of inhospital mortality were identified using patient diagnoses, demographic characteristics, and basic clinical and laboratory data from those admitted to the CCU^11^.

In our study, we used the comprehensive data of the MORCOR-TURK trial and calculated the GPRs of patients on CCU admission.

### Ethical considerations

The study protocol was reviewed and approved by the Ethics Committee of Afyonkarahisar University of Health Sciences, Afyon, Turkey (No: 2022/9-422; Date: August 5, 2022). Written informed consent was obtained from all participants or their relatives.

### Study population

Eligible participants for the study were individuals aged 18 years or older who experienced cardiac emergencies. Exclusions included noncardiac admissions, discharges within 4 h at the patient's request, admissions after elective procedures, and admissions under cardiopulmonary resuscitation without response. Data collection involved demographic and clinical information, hemodynamic status, laboratory results, primary diagnoses, inhospital events, and discharge status. Participants received standard care under the supervision of a cardiologist, including medical and interventional treatment. Medications, vital signs, and adverse events were recorded, such as arrhythmias, strokes, renal failure, bleeding, and mortality. No additional medical interventions were administered as part of the study^12^.

### Statistical analyses

The R-based statistical package JAMOVI program was used for the analysis. After 50 CCU centers were identified as participants in the study, stratified sampling, along with the random sampling method, was used according to the weights of the centers. The power analysis of the study was performed using G Power 3.1.9.2. software (Universität Kiel, Germany). Considering an α type error rate of 0.005 and a power of 0.95, in addition to an approximately 10% mortality rate in the literature, at least 1,092 participants needed to be included in the study (effect size was 0.5).

Two-sided p<0.05 was taken for statistical significance. Data were expressed as mean and standard deviation. The Kolmogorov-Smirnov test examined the normality distribution of variables, and Levene's test assessed the homogeneity of variances. Mean±standard deviation, interquartile ranges, and percentage schemes were used for normally distributed continuous variables, non-normally distributed variables, and categorical variables, respectively. Two-tailed Student's t-test and the Mann-Whitney U test were used for parameters that were normally distributed and non-normally distributed; the chi-square test and Fisher's exact test were used for categorical variables.

Candidate predictors for primary outcome should be clinical and statistically plausible. We considered variables under the mentioned principles. Hence, predictors [age, sex, mean blood pressure, creatinine, C-reactive protein (CRP), white blood cell (WBC), and GPR] were evaluated by univariate and multivariable logistic regression (LR) analyses. To evaluate the impact of GPR on mortality, two models were created. Model 1 considered age, heart failure history, chronic kidney disease, hypertension, diabetes mellitus, and coronary artery disease. Model 2 included GPR in addition to Model 1. The models were compared using −2 log-likelihood ratio, Nagelkerke R2, and area under the curve (AUC). A p-value less than 0.05 indicated statistical significance in all analyses.

## RESULTS

The study included 3,157 patients with a mean age of 65 years (range: 56–73), of whom 2,087 (66.1%) were male. Arterial hypertension was diagnosed in 1,864 patients (59%), diabetes mellitus in 1,184 patients (37.5%), hyperlipidemia in 1,120 patients (35.5%), and smoking in 1,093 patients (34.6%). Other common diagnoses included decompensated HF with 339 patients (10.7%) and arrhythmia with 272 patients (8.6%) [12]. Table 1 provides an overview of the baseline characteristics, comorbidities, and laboratory findings of the study group. Notably, several laboratory parameters exhibited statistically significant differences in the nonsurvivor group. These included glucose [151 (114–212) vs 123 (101–164); p<0.001], potassium (K) (4.7±0.8 vs 4.4±0.6; p<0.001), CRP [23.6 (10–120) vs 5.7 (1.9–16); p<0.001], WBC count (12.3±5.3 vs 9.9±3.5; p<0.001), neutrophil count (9.3±4.8 vs 7.0±3.3; p<0.001), and GPR (47.0±22.2 vs 35.7±18.7; p<0.001).

**Table 1:** Baseline characteristics of the study group.

	All patients (n=3,157)	Nonsurvivors (n=137)	Survivors (n=3,020)	p-value
Age (years)	65 (56–73)	73 (63–83)	65 (56–73)	<0.001
Male gender, n (%)	2,087 (66.1)	73 (53.3)	2,014 (66.7)	0.002
Hypertension, n (%)	1,864 (59)	91 (66.4)	1,773 (58.7)	0.076
Diabetes mellitus, n (%)	1,184 (37.5)	55 (40.1)	1,129 (37.4)	0.528
Dyslipidemia, n (%)	1,120 (35.5)	46 (33.6)	1,074 (35.6)	0.715
Smoking, n (%)	1,179 (61.9)	79 (57.6)	1,876 (62.1)	0.043
Family history of CAD, n (%)	1,101 (34.9)	37 (27.2)	1,064 (35.8)	0.043
CAD, n (%)	1,446 (45.8)	74 (54)	1,372 (45.4)	0.054
PAD, n (%)	122 (3.9)	6 (4.4)	116 (3.8)	0.005
AF, n (%)	483 (15.3)	34 (24.8)	449 (14.9)	0.004
Heart failure, n (%)	978 (31)	76 (55.5)	902 (29.9)	<0.001
Stroke history, n (%)	142 (4.5)	11 (8)	131 (4.3)	0.086
Glucose, mg/dL (Median, IQR)	124 (102–166)	151 (114–212)	123 (101–164)	<0.001
Creatinine mg/dL (Median, IQR)	1.2 (0.3–9.8)	1.7 (0,3–6,5)	1.1 (0.3–9.8)	<0.001
GFR, mL/min (Median, IQR)	78 (54–95)	39 (22.7–78)	79 (57–95)	<0.001
Na, mg/dL (Mean ± SD)	137.6 ± 4.0	137.3 ± 5.7	137.6 ± 3.9	0.324
K, mg/dL (Mean ± SD)	4.4 ± 0.6	4.7 ± 0.8	4.4 ± 0.6	<0.001
ALT, mg/dL (Median, IQR)	20 (14–32)	24 (12–73)	20 (14–32)	0.001
CRP, mg/dL (Median, IQR)	6 (2–17)	23.6 (10–120)	5.7 (1.9–16)	<0.001
WBC, mg/dL (Mean± SD)	10.0 ± 3.7	12.3 ± 5.3	9.9 ± 3.5	<0.001
Hemoglobin, mg/dL (Mean ± SD)	13.3 ± 2.2	11.9 ± 2.3	13.3 ± 2.2	<0.001
GPR (Mean ± SD)	35.2 ± 18.4	47.0 ± 22.2	35.7 ± 18.7	<0.001

CAD: coronary artery disease; PAD: peripheral artery disease; AF: atrial fibrillation; GFR: glomerular filtration rate; Na: sodium; K: potassium; ALT: alanine aminotransferase; CRP: C-reactive protein; WBC: white blood cell; GPR: glucose potassium ratio; SD: standard deviation.

**Figure 1:**
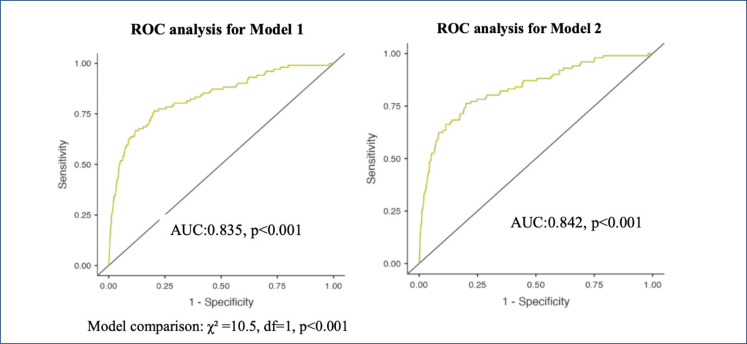
Comparison of the predicted models' receiver operating characteristic analysis.

As shown in Table 2, our multivariable LR analyses belonging to Model 2 revealed that age (OR 1.031, 95%CI 1.011–1.051, p<0.001), female sex (OR 1.960, 95%CI 1.242–3.093, p<0.001), mean blood pressure (OR 0.943, 95%CI 0.930–0.95, p<0.001), creatinine levels (OR 1.477, 95%CI 1.230–1.774, p<0.001), CRP (OR 1.005, 95%CI 1.002–1.009, p=0.002), WBC (OR 1.079, 95%CI 1.028–1.133, p=0.002), and GPR (OR 1.015, 95%CI 1.006–1.024, p<0.001) independently correlated with mortality. Model 1 and Model 2 are compared to determine the effect of GPR on mortality and to test the discrimination ability of the created model using the −2 log-likelihood ratio, Nagelkerke R2, and AUC. In Model 1, the −2 log-likelihood ratio was 1,050, Nagelkerke R^2^ was 0.254, and AUC was 0.835 (95%CI 0.793–0.889, p<0.001). In Model 2 after GPR is added, the −2 log-likelihood ratio was 911, Nagelkerke R^2^ was 0.268, and AUC was 0.842 (95%CI 0.808–0.877, p<0.001) (Figure 1).

**Table 2:** Comparison of multivariate logistic regression models for the mortality in coronary care unit.

	Model 1			Model 2		
	OR	95%CI	p-value	OR	95%CI	p-value
Age	1.029	1.010–1.048	0.003	1.031	1.011–1.051	0.002
Sex (female)	2.038	1.298–3.198	0.002	1.960	1.242–3.093	0.004
Mean BP	0.942	0.928–0.955	<0.001	0.943	0.930–0.957	<0.001
Creatinine	1.457	1.216–1.746	<0.001	1.477	1.230–1.774	<0.001
CRP	1.006	1.002–1.009	<0.001	1.005	1.002–1.009	0.002
WBC	1.090	1.039–1.143	<0.001	1.079	1.028–1.133	0.002
GPR	–	–	–	1.015	1.006–1.024	<0.001

OR: odds ratio; CI: confidence interval; BP: blood pressure; CRP: C-reactive protein; WBC: white blood cell; GPR: glucose potassium ratio. R^2^_N_ (Nagelkerke's R) for Model 1=0.254; R^2^_N_ for Model 2=0.268.

According to the receiver operating characteristic (ROC) analysis, the AUC value of Model 2 was statistically higher than Model 1 (model comparison: χ^2^=10.5, df=1, p<0.001) (Figure 1). A statistically significant correlation was found between death and GPR in Model 2 (OR 1.015, 95%CI 1.006–1.024, p<0.001).

## DISCUSSION

To the best of our knowledge, this is the first study to investigate the correlation between the serum GPR and mortality in patients hospitalized in CCU. The study results demonstrate that the serum GPR is significantly associated with the inhospital mortality of CCU patients.

Stress hyperglycemia is a temporary rise in blood glucose levels triggered by increased glucagon secretion during acute physiological stress. It is a survival response to stress and can occur regardless of the presence of diabetes. The body's response to hyperglycemia involves several metabolic hormones, including glucagon, epinephrine, and growth hormone. Stress can lead to insulin deficiency and insulin resistance, resulting in high blood glucose levels and reduced insulin sensitivity. Additionally, hyperglycemia has been shown to increase the likelihood of cellular dysfunction and apoptosis^13–16^. Due to these mechanisms, stress-induced hyperglycemia is associated with an increased risk of malignant events, inhospital mortality, and poor prognosis in patients with cardiovascular disease^16^.

The severity of the disease has been shown to be associated with excessive release of catecholamines, leading to sympathetic activation in CCU patients. The production of large amounts of catecholamines, oxidative stress response, and insulin resistance may increase the levels of free fatty acids in the body, which have a toxic effect on infarcted and ischemic myocardium^17^. This adversely affects the prognosis of patients admitted to CCU^18^. Hospital mortality rates increase when serum glucose levels exceed 110–120 mg/dL in nondiabetic patients and 200 mg/dL in diabetic patients^19^.

Potassium ions are the most abundant cations in human body cells and play a crucial role in various physiological processes, such as nerve conduction, heartbeat, muscle contraction, and maintenance of normal kidney function. The active Na+-K+-ATPase pump is responsible for transporting potassium across the cell membrane. Catecholamines released in response to stress can affect the function of the Na+-K+-ATPase pump, leading to a decrease in serum potassium levels^4^.

These findings suggest that GPR has clinical significance in the prognostic evaluation of patients hospitalized in CCU with diagnoses such as ACS, acute decompensated heart failure, and cardiac arrhythmias that activate stress response in the body. In their study of 17,670 acute myocardial infarction patients, Plakht et al. found that the co-occurrence of low potassium and high glucose levels was the highest independent risk factor for inhospital mortality^20^.

The serum GPR is a novel parameter that can be rapidly measured in clinics and has been used in a few studies due to the potential combined effects of glucose and serum potassium. Studies have shown that GPR levels are associated with prognosis and mortality in various clinical situations, including acute neurological injury, neuropsychiatric syndrome following carbon monoxide poisoning, intracerebral hemorrhage after severe traumatic brain injury, and ischemic stroke^6–10^. In each of these studies, patients with high GPR had significantly higher mortality. In a recent study, it is also demonstrated that GPR can be useful for diagnosing massive pulmonary embolism from non-massive pulmonary embolism^21^.

GPR may help to predict the prognosis of CCU patients in the early stage. According to our results, the administration of appropriate potassium replacement, hypoglycemic treatment, and planned treatment to control the stress response (e.g., β-blockers and renin-angiotensin-aldosterone system (RAAS) inhibitors) may improve inhospital mortality and prognosis in patients with high GPR levels in CCUs.

One potential limitation of our study is that mortality rates may be influenced by the interventional facilities of the centers, as well as the competence and experience of the operators. Another limitation is its cross-sectional and observational design, which may introduce bias and confounding.

## CONCLUSION

In CCU patients, there is a positive linear correlation between GPR and inhospital mortality. GPR has the potential to be a simple and rapid predictor of inhospital mortality in CCU patients.

## ETHICAL CONSIDERATIONS

The study protocol was reviewed and approved by the Ethics Committee of Afyonkarahisar University of Health Sciences, Afyon, Turkey (No: 2022/9-422; Date: August 5, 2022).
